# Whole-Exome Sequencing of Nasopharyngeal Carcinoma Families Reveals Novel Variants Potentially Involved in Nasopharyngeal Carcinoma

**DOI:** 10.1038/s41598-019-46137-4

**Published:** 2019-07-09

**Authors:** Guoqin Yu, Wan-Lun Hsu, Anna E. Coghill, Kelly J. Yu, Cheng-Ping Wang, Pei-Jen Lou, Zhiwei Liu, Kristie Jones, Aurelie Vogt, Mingyi Wang, Sam M. Mbulaiteye, Hao-Hui Chen, Joseph Boland, Meredith Yeager, Scott R. Diehl, Chien-Jen Chen, Allan Hildesheim, Alisa M. Goldstein

**Affiliations:** 1 Integrative Tumor Epidemiology Branch, Division of Cancer Epidemiology and Genetics, National Cancer Institute, Bethesda, MD USA 20892; 2 Genomics Research Center, Academia Sinica, and Graduate Institute of Epidemiology and Preventive Medicine, College of Public Health, National Taiwan University, Taipei, Taiwan; 3 Infections and Immunoepidemiology Branch, Division of Cancer Epidemiology and Genetics, National Cancer Institute, Bethesda, MD USA 20892; 4 Department of Otolaryngology, National Taiwan University Hospital, National Taiwan University, Taipei, Taiwan; 5 Cancer Genomics Research Laboratory, Division of Cancer Epidemiology and Genetics, National Cancer Institute, Bethesda, MD 20892 and LEIDOS, Frederick, MD USA; 6 Rutgers School of Dental Medicine, Newark, NJ USA; 7 Clinical Genetics Branch, Division of Cancer Epidemiology and Genetics, National Cancer Institute, Bethesda, MD USA 20892

**Keywords:** Cancer genetics, Head and neck cancer

## Abstract

Genetic susceptibility is likely involved in nasopharyngeal carcinoma (NPC), a cancer caused by Epstein-Barr virus (EBV) infection. Understanding of genetic factors involved in NPC and how they contribute to EBV-induced carcinogenesis is limited. We conducted whole-exome capture/sequencing among 251 individuals from 97 multiplex families from Taiwan (205 affected, 21 obligate carriers, and 25 unaffected) using SeqCap EZ Human Exome Library v3.0 and Illumina HiSeq. Aligned sequences were filtered to identify likely-to-be-functional deleterious variants that co-segregated with disease. Ingenuity Pathway analysis was performed. Circulating magnesium levels were measured in 13 individuals in 2 families with NIPAL1 mutations and in 197 sporadic NPC cases and 237 controls. We identified variants in 12 genes likely involved in cancer pathogenesis, viral infection or immune responses to infection. These included genes postulated to be involved in magnesium transport (NIPAL1), EBV cell entry (ITGB6), modulation of EBV infection (BCL2L12, NEDD4L), telomere biology (CLPTM1L, BRD2, HNRNPU), modulation of cAMP signaling (RAPGEF3), DNA repair (PRKDC, MLH1), and Notch signaling (NOTCH1, DLL3). Pathway based analysis demonstrated enrichment for Notch signaling genes (p-value = 0.0006). Evaluation of individuals within NIPAL1 families suggested lower serum magnesium in NPC compared to unaffected members. A significant reduction in serum magnesium levels was observed among sporadic NPC cases compared to controls (7.1% NPC/1.7% controls below normal range; OR = 4.5; 95% CI = 1.4,14) and is consistent with findings demonstrating a role for magnesium channeling in T-cell responses to EBV. We identified novel genes associated with NPC that point to new areas of inquiry to better understand genetic factors that determine the fate of viral infections and/or otherwise predisposes to NPC.

## Introduction

Nasopharyngeal carcinoma (NPC) is an epithelial tumor caused by Epstein-Barr virus (EBV), an infection that typically occurs in childhood or adolescence and persists lifelong^1^. An intriguing feature of NPC is that while the infection that causes this cancer (EBV) is nearly ubiquitous worldwide (>90% adults infected) and a necessary cause for undifferentiated forms of NPC that account for over 90% of all NPC cases in high-risk regions, the cancer itself occurs much less frequently and with a geographic distribution that is strikingly uneven^1^. This suggests that factors other than EBV are important determinants of which individuals develop NPC. An important role for host genetic susceptibility is suggested by (1) the strong predilection of this disease for individuals of particular ethnicities (i.e., individuals of Chinese, Inuit and Berber descent), (2) migrant studies showing residual elevated incidence in second and third generation migrants from high-risk to low-risk regions, (3) observed familial clustering of NPC in endemic regions, and (4) epidemiological studies showing that a family history of NPC is associated with a 4-8 fold increased risk of disease^1^. The study of NPC therefore provides a unique opportunity to elucidate the interplay between oncogenic infections and host genetics in cancer etiology.

Genome-wide association studies (GWAS) of sporadic NPC have reproducibly highlighted the link between this cancer invariably caused by EBV and variation in the MHC region on chromosome 6^2,3^. These results support decades of research demonstrating a role for HLA genes (genes located within the MHC region) and NPC, likely via differential ability of distinct HLA alleles to effectively trigger an immune response targeting EBV infection via presentation of viral antigens to cytotoxic T-cells^4,5^. However, while findings regarding the association between HLA genes and NPC have been strong and consistent, polymorphisms in HLA genes only explain a minority of NPC cases and other genetic factors are therefore likely to be involved. These additional host genetic susceptibility factors, however, have remained largely elusive.

One meta-analysis of four NPC GWAS followed by a replication effort recently reported a significant modest (~20% increase in risk) association between SNPs located within the CLPTM1L/TERT, MECOM, TNFRSF19, and CDKN2A/B gene regions and NPC^6^. In addition to studies of sporadic NPC, studies of familial NPC have identified chromosomal regions linked to NPC development, but findings have differed across studies and the genes involved remain unknown^7^. Recent whole exome sequencing (WES) studies of NPC have identified additional genes that might be important in NPC development, including *KMT2C* and *MST1R*^8,9^. Taken together, these findings highlight the complex nature of genetic susceptibility factors linked to NPC development.

To further understand genetic susceptibility factors involved in NPC development and how inherited genetic factors might contribute to NPC, we conducted a large-scale NPC multiplex family study followed by whole exome sequencing (WES). WES was performed on 251 individuals from 97 informative NPC multiplex families. We report the identification of novel inherited genetic variants that segregate with NPC within multiplex families. Some variants potentially operate via biological pathways that might impact EBV infectivity and/or immune control or are modulated by oncogenic EBV infection. Other novel variants involve genes in telomere maintenance and DNA repair, confirming the likely role of these biological pathways in NPC development.

## Results

### Characteristics of population

Results from 251 individuals from 97 NPC families were included in this analysis. NPC cases were 43.1 years (range: 18–80) at diagnosis, on average. Obligate carriers and unaffected family members were, on average, 68.0 (range: 47–91) and 67.6 (range: 53–87) years, respectively. 69.8% of cases were male. The corresponding percentage for obligate carriers and unaffected family members was 52.4% and 36.0%.

### Whole exome sequencing implicates variants in genes involved in magnesium transport, EBV entry into epithelial cells, modulation of EBV infection, telomere biology, DNA repair, and notch signaling

We identified potential rare variants in 608 genes and present these genes in Supplemental Table 2. After consideration of evidence from the literature indicative that the variation occurs in genes involved in cancer, viral infection or immune responses, 12 genes were flagged as being most plausibly involved in NPC pathogenesis. Table 1 summarizes the 13 co-segregating variants with CADD > 15 from these 12 genes that met the family-based criteria described in the methods. For completeness, Supplemental Table 3 shows all rare nonsynonymous/loss-of-function variants observed in these 12 genes. Supplemental Table 3 includes variants for these 12 genes that did not meet the CADD or family co-segregation criteria required for variants shown in Table 1. Table 1 and Supplemental Table 3 include genes involved or postulated to be involved in magnesium transport (NIPAL1), EBV entry into epithelial cells (ITGB6), modulation of EBV infection via LMP1 binding (BCL2L12, NEDD4L), telomere biology (CLPTM1L, BRD2, HNRNPU), modulation of cAMP signaling (RAPGEF3), DNA repair (PRKDC, MLH1), and Notch signaling (NOTCH1, DLL3). Three genes had co-segregating variants in single families while 9 genes (RAPGEF3, NIPAL1, PRKDC, BCL2L12, HNRNPU, BRD2, MLH1, NOTCH1, and ITGB6) had co-segregating variants in 2-4 families (Table 1 and Supplemental Table 3). For genes with variants identified in multiple families, the changes observed include nonsynonymous changes plus 3 nonframeshift deletions (RAPGEF3, NOTCH1, and HNRNPU), and one splice variant (NIPAL1). Predicted protein changes for genes with variants identified in a single family were all nonsynonymous.

**Table 1:** Summary of Top Candidate Genes with Rare, Likely Deleterious Variants Linked to NPC Identified in the Taiwan NPC Family Whole Exome Sequencing Study.

Gene Name	Chromosome	Position	Reference	Variant	Change Observed	ID of Family(ies) Where Variant Observed	No. of Affected/ Obligate Carriers/Unaffected Sequenced^a^
**Genes/Variants Prioritized Based on Literature Review**							
BCL2L12	chr19	50169135	C	T	Nonsynonymous	6016, 4061	4/0/0
BRD2	chr6	32948153	A	T	Nonsynonymous	4105, 1003, 3002	6/0/0
CLPTM1L	chr5	1320735	G	A	Nonsynonymous	6014	2/1/0
DLL3	chr19	39991273	A	G	Nonsynonymous	4013	3/0/0
HNRNPU	chr1	245021539	T	C	Nonsynonymous	3050, 4060, 5078	6/0/0
ITGB6	chr2	160993948	C	T	Nonsynonymous	5013	2/0/1
MLH1	chr3	37067192	C	T	Nonsynonymous	3001	2/1/1
NEDD4L	chr18	56002735	G	A	Nonsynonymous	3033	2/0/1
NIPAL1	chr4	48032135	A	G	Splice Variant	4082	4/0/0
NOTCH1	chr9	139399922	C	T	Nonsynonymous	5098	2/1/0
PRKDC	chr8	48749063	C	T	Nonsynonymous	6014	2/1/0
RAPGEF3	chr12	48131365	TCGGGAGAGG	T	Nonframeshift Deletion	5098	2/1/0
RAPGEF3	chr12	48143184	G	A	Nonsynonymous	1062	2/1/2
**Additional Genes/Variants Prioritized Based on IPA Pathway Analysis**							
LFNG	chr7	2565127	C	T	Nonsynonymous	6014	2/1/0
MAML1	chr5	179198178	C	T	Nonsynonymous	3055	2/0/0
MAML1	chr5	179192654	G	C	Nonsynonymous	4084	2/0/0
MFNG	chr22	37875518	G	A	Nonsynonymous	3063	2/0/0
MFNG	chr22	37875510	T	G	Nonsynonymous	4113	2/0/0
PSEN2	chr1	227077760	A	G	Nonsynonymous	3002	2/0/0
PSEN2	chr1	227075798	C	A	Nonsynonymous	5092	2/0/0

^a^Variant was observed in affected individuals/obligate carriers sequenced; not observed in unaffected individuals sequenced.

### Pathway based analysis suggest enrichment for variations in genes involved in Notch signaling

To evaluate all candidate genes identified, pathway based analysis of genes included in Supplemental Table 2 was performed and demonstrated considerable enrichment for variation in genes involved in Notch signaling (6 of 38 genes or 16%; p-value 0.0006) (Table 2). The co-segregating variants with CADD > 15 observed within genes in the Notch signaling pathway are summarized in Table 1 and include 9 variants in the following 6 genes: NOTCH1 (single variant), DLL3 (single variant), LFNG (single variant), MAML1 (2 variants), MFNG (2 variants), PSEN2 (2 variants). Additional rare nonsynonymous variants in these genes are included in Supplemental Table 3. Suggestive evidence for enrichment was noted for the following pathways: epithelial adherens junction signaling (p-value 0.0025), apoptosis signaling (p-value 0.0073), nNOS signaling in skeletal muscle cells (p-value 0.0091), and nNOS signaling in neurons (p-value 0.0095).

**Table 2:** Summary of Most Enriched Canonical Pathways Based on Ingenuity Pathway Analysis (IPA).

Ingenuity Canonical Pathways	Which Evidence for Rare Variants Were Observed (n/N; %)	p-value	Genes within Pathway for Which Rare Variants Were Observed
Notch Signaling	6/38 (16%)	0.0006	MAML1,MFNG,LFNG, PSEN2,DLL3,NOTCH1
Epithelial Adherens Junction Signaling	11/146 (8%)	0.0025	MET,MYH10,CDH2,MYH8,LEF1,JUP,PARD3,IQGAP1,NOTCH1,FARP2,MYH1
Apoptosis Signaling	8/89 (9%)	0.0073	GAS2,IKBKB,APAF1,CAPN9,CAPN2,SPTAN1,CAPN3,BIRC2
nNOS Signaling in Skeletal Muscle Cells	3/15 (20%)	0.0091	NOS1,RYR3,CAPN3
nNOS Signaling in Neurons	5/47 (11%)	0.0095	NOS1,CAPN9,CAPN2,PRKD3,CAPN3

When the cBioPortal and Cosmic public databases were queried, we identified evidence for alterations in NOTCH1 (one truncating and one missense mutations) in 4% of the 56 tumors evaluated in a study conducted in Singapore^10^.

### Examination of 20 variants in Table 1 in publicly available NPC WES datasets

We evaluated the 20 germline variants in Table 1 using two publicly available NPC WES datasets. These datasets included 39 early-age-of-onset NPC samples from Dai *et al*.^8^ and 56 NPC germline/normal samples from Lin *et al.*^10^. Overall, we observed three variants from Table 1 in the two datasets of NPC cases. We observed two variants in single NPC cases from the Lin *et al*. study. This included one NPC case with a missense variant in PSEN2 (p.His169Asn) and a second NPC case with the missense variant in CLPTM1L (p.Arg510Trp); this latter variant was observed only once in gnomAD with a frequency of 0.00003. Finally, we observed the missense variant in BRD2 (p.Glu762Asp) in one NPC case from the study of Dai *et al*.^8^.

### Variants in *KMT2C* and *MST1R*

NPC families with at least two NPC cases/obligate carriers sequenced were examined for rare variants in two postulated NPC susceptibility genes KMT2C and MST1R. Supplemental Table 4 shows the rare variants found in this sample using the same allele frequency filtering criteria as used for the primary analyses (see criteria in Methods). Although multiple rare variants in both KMT2C and MST1R were observed, only one variant in MST1R fully co-segregated with disease in a family with >2 NPC cases/obligate carriers sequenced. This variant was not, however, predicted to be deleterious according to CADD (8.943) and occurred in East Asian non-cancer subjects from ExAC at an allele frequency of 0.0017.

### Reduced levels of magnesium observed in NPC cases/obligate carriers from families with NIPAL1-containing variants and in sporadic NPC cases

**Figure 1:**
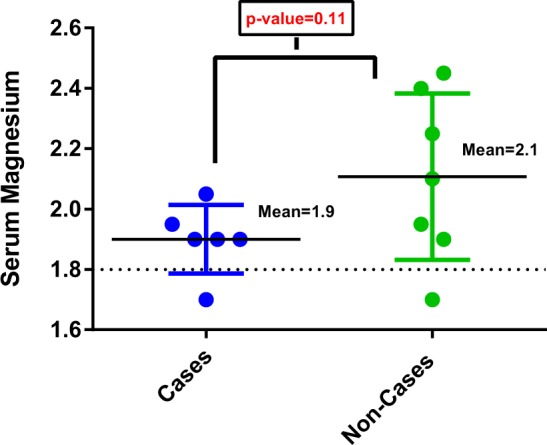
Legend: Magnesium levels among NPC cases and unaffected family members from two NPC multiplex families with NIPAL1 mutations.

The variants identified in the NIPAL1 gene in two families (Table 1 and Supplemental Table 3) were intriguing because NIPAL1 is involved in magnesium channeling and studies have demonstrated that another gene involved in such channeling (MagT) is responsible for a rare primary immunodeficiency condition called “X-linked immunodeficiency with magnesium defect, EBV infection, and neoplasia” (XMEN), which predisposes carriers to uncontrolled EBV infection due to an inability to activate T-cells in response to EBV infection^11^. We therefore explored our NIPAL1 findings further by testing family members within the 2 families with NIPAL1 variants for total serum magnesium levels, and by evaluating the association between total serum magnesium levels and sporadic NPC using routinely available pre-treatment magnesium testing results from NPC cases and non-cases attending the ENT clinic at the National Taiwan University Hospital. We observed reduced, albeit not statistically significant, serum magnesium levels among the 6 NPC cases from our families with NIPAL1 variants compared to 7 unaffected individuals from these same families (1.9 mg/dL vs. 2.1 mg/dL; p-value 0.11) (Fig. 1). Among unaffected individuals, magnesium levels were not significantly different between variant carriers (N = 3; 2.2 mg/dL) than non-carriers (N = 4; 2.0 mg/dL; p-value 0.41). Consistent with the differences observed between affected and unaffected individuals in our family study, an independent set of sporadic NPC cases (N = 197; 7.1% below normal range) were 4.5 (95% CI = 1.4–14; p-value = 0.0065) times more likely than non-NPC controls (N = 237; 1.7% below normal range) to have clinical serum magnesium deficiency prior to treatment initiation. This effect remained in analyses that restricted NPC cases to those diagnosed with early stage (stages I/II) disease (OR = 5.3; 95% CI = 1.6–18; p-value = 0.008).

## Discussion

We performed a WES study within a large collection of families with multiple individuals affected with NPC to identify novel inherited variants involved in the development of this disease. Because infection with EBV is necessary for NPC, our study also afforded the opportunity to learn about genetic factors that are potentially involved in the modulation of EBV and thereby gain insights into virus-host interactions important for carcinogenesis.

Whole exome sequencing of 251 individuals from 97 NPC families led to the identification of numerous rare variants possibly linked to NPC, including variants within genes involved in Notch signaling (NOTCH1, DLL3), magnesium transport (NIPAL1), EBV entry into epithelial cells (ITGB6), modulation of EBV (BCL2L12, NEDD4L), telomere biology (CLPTM1L, BRD2, HNRNPU), modulation of cAMP signaling (RAPGEF3), or DNA repair (PRKDC, MLH1). Pathway based analysis provided support for a role in NPC pathogenesis for additional genes in the Notch signaling pathway. Finally, direct testing for circulating magnesium levels in individuals within our families and sporadic NPC cases and controls provided support for the role of magnesium transport genes such as NIPAL1 in NPC. These findings add to the known list of candidate genetic susceptibility factors linked to familial NPC that previously included polymorphisms in HLA and selected other genes^12–15^.

We identified rare variants potentially linked to NPC within 6 genes involved in Notch signaling, including variants in NOTCH1 and in an extracellular Notch ligand, DLL3. This is consistent with a report from a previous study of 56 sporadic NPC cases in which alterations in NOTCH1 were noted for 4% of cases evaluated^10^. Notch signaling is an evolutionarily conserved pathway known to play an important role in cell differentiation, proliferation and apoptosis^16,17^. Extensive work has linked Notch and Notch signaling genes to both hematopoietic and solid tumors^16,17^. Of note, EBV, the virus linked to NPC development, encodes a functional homologue of intracellular notch (EBNA2) that has been shown to act as a constitutively activated form of Notch^18–20^. EBNA2 is known to be critical in initial EBV infection and necessary for EBV-transformation of infected B cells, suggesting an important role for modulation of Notch signaling in establishment of the lifelong viral infections linked to subsequent NPC development^18,19^. Our results suggest that inherited changes in Notch and other genes in the Notch pathway may predispose to the development of NPC and one might speculate that such a link is due to modulation of its viral cause, EBV infection.

We also identified rare variants potentially linked to NPC within a magnesium transport gene, NIPAL1. We further showed evidence suggesting that circulating magnesium levels are reduced in individuals with NPC, both in the context of NPC multiplex families who carry NIPAL1 variants and of sporadic NPC. While our finding of reduced circulating magnesium levels in sporadic NPC cases compared to controls does not directly implicate NIPAL1, our findings take on added significance when considered in light of the recent discovery that null mutations in another magnesium transport gene, MAGT1, which abrogates magnesium flux in NK and CD8 T cells, predisposes to uncontrolled EBV infection and EBV-associated lymphomas^11,21^. We did not observe evidence for rare mutations in MAGT1 in the NPC families included in our study. Taken together, these findings suggest that genes that regulate magnesium transport across cells might be important determinants of NPC risk and one potential explanation for such a link is that these genes are important determinants of a host’s ability (or lack thereof) to mount an effective immune response against EBV, the virus linked to NPC.

Rare variants within the ITGB6 gene that codes for an integrin were also identified in our NPC families. Integrins are transmembrane proteins expressed in epithelial cells that are important for cell-to-cell and cell–to–extracellular matrix communications^22^. In the context of EBV and NPC pathogenesis, integrins are of relevance because studies suggest that they are important for EBV anchoring/fusion to epithelial cells, thus facilitating its entry into and infection of epithelial cells^23,24^. Given the role of integrins in EBV infection of epithelial cells, variants in genes that code for these proteins could modulate efficiency of viral-cell interactions that lead to infection of epithelial cells and yield epithelial cells exposed to EBV virions (such as those at the nasopharynx) either more or less susceptible to EBV infection and therefore more or less susceptible to the development of EBV-associated epithelial cancers such as NPC. Future studies aimed at understanding the effect of variants in ITGB6 and other integrin-coding genes on EBV infectivity could shed light on the specific mechanism(s) that explain our current findings.

Additional genes coding for protein with which EBV interacts and that we found to contain rare variants that segregate with disease in our NPC families include BCL2L12 and NEDD4L. These genes code for protein products that are known to interact with LMP1, an EBV protein that functions as a viral oncogene via interactions with BCL2^25–28^. Interestingly, LMP1 has been shown to be expressed frequently in NPC tumors^29^.

We also identified rare variants within genes that have previously been shown to be involved in processes important for tumorigenesis (independent of EBV infection). These include genes involved in DNA repair (PRKDC, MLH1) and telomere biology (CLPTM1L, BRD2, HNRNPU). With respect to the latter, it is noteworthy that a recent meta-analysis of NPC GWAS and replication study implicated polymorphisms in CLPTM1L/TERT region with NPC^6^. Our findings therefore support a role for telomere maintenance and its regulation and DNA repair in NPC pathogenesis.

After completion of our analyses, two WES studies of NPC cases and controls were identified based on published work^8,9^. Although not definitive, *KMT2C* and *MST1R* were proposed as candidate susceptibility genes for NPC. We, therefore, reviewed the families in the current study with at least two NPC cases/obligate carriers sequenced for rare variants in these two candidate genes. Although multiple rare variants were observed, only one variant in *MST1R* fully co-segregated with NPC in a family with >2 NPC cases/obligate carriers sequenced. This observed missense variant produced a protein change (p.G356D) that was not predicted to be deleterious in any in silico program reviewed including SIFT, PolyPhen, FATHMM, Mutation Taster, Mutation Assessor, Provean, LRT, or CADD. In addition, this variant was observed in East Asian non-cancer subjects from ExAC with a relatively high frequency of 0.0017 suggesting that it is likely not disease-related. The *MST1R* variant reported by Dai *et al*.^8^ that was most strongly associated with NPC (c.G917A:p.R306H) was not observed in any of the 97 NPC families in the current study. In summary, there was little additional supporting evidence for either proposed NPC candidate gene.

We searched for publicly available NPC WES datasets to further investigate variants identified in the multiplex NPC families. We conducted variant calling (see methods) for two WES datasets that included 39 early-age-of-onset NPC samples from Dai *et al*.^8^ and 56 NPC germline/normal samples from Lin *et al*.^10^ and evaluated the 20 germline variants reported in Table 1. Although we observed single NPC cases with variants in PSEN2 (p.His169Asn), BRD2 (p.Glu762Asp), and CLPTM1L (p.Arg510Trp), there was insufficient evidence for further support for any of the candidate genes/variants given the small sample sizes of these publicly available NPC WES datasets.

Our study is not without limitations. One limitation of exome sequencing studies such as ours, is the limited ability to define precise mechanisms for our findings. While our findings, particularly those for genes involved in EBV infection, response and life cycle are suggestive, future laboratory studies will be required to further elucidate their biological functions and relationship to tumorigenesis for EBV-associated cancers. Another limitation of our work is the fact that all variants identified occurred in a small number of families, only a subset of which were observed in all cases/obligate carriers within the families, suggesting that they are unlikely to explain a large fraction of NPC. Nonetheless, they point to important genes potentially involved in NPC development that might suggest avenues for future investigation aimed at identifying additional, more common changes (genetic or epigenetic) in these genes/pathways that might explain a larger fraction of NPC cases. Finally, by design, we targeted the identification of rare genetic alterations with a dominant mode of inheritance. Therefore, alterations that operate in a non-dominant fashion (e.g., recessive trait) might have been missed by our study.

Strengths of our study include the fact that it is embedded within the largest study of NPC multiplex families conducted to date, that a sizeable number of individuals were sequenced within these families, and that the sampling frame for identifying our multiplex families relied on a long standing nationwide cancer registry maximizing the representativeness of the NPC multiplex families evaluated. Our ability to follow-up findings for NIPAL1 by demonstrating reductions in magnesium levels in familial and sporadic NPC is also a strength of our work.

## Conclusions

Results from this study implicate variation in numerous genes involved in Notch signaling, magnesium transport, EBV entry into epithelial cells, modulation of EBV infection, telomere biology, and DNA repair in the development of NPC. Our findings with respect to magnesium transport genes, which may be important for activation of T-cells in response to EBV infection, are of particular note, given our independent finding of reduced magnesium levels among NPC cases. Our findings pave the way for future work to better understand genetic factors that are important in response to viral infections and/or otherwise predispose to virus-associated cancers.

## Materials and Methods

### Study population

Participants were part of the previously described cancer registry based NPC Multiplex Family Study in Taiwan^30–33^. Briefly, 20,450 NPC cases diagnosed between 1980 and 2003 were identified through the National Cancer Registry, 10 tertiary care hospitals and select outpatient clinics that treat NPC. As in other NPC endemic areas, over 90% of these cases are expected to be of histological types linked to EBV infection^1^. We successfully screened 10,178 (49.8%) cases for a family history of NPC. Ultimately, 358 NPC multiplex families were identified and recruited into our study. From these families, a total of 3,216 individuals (659 NPC cases and 2,557 unaffected family members) were included in the parent study, consisting of NPC cases, their parents, up to 5 siblings, and for deceased NPC cases, their spouse and up to 3 children. When it was necessary to link affected individuals in our families, additional relatives were recruited. Blood (in acid citrate dextrose buffer vacutainers) from which DNA was extracted using a salt precipitation method was obtained from participants and stored at −80 °C. Subjects provided informed consent and the protocol was reviewed/approved by human subjects review committees in Taiwan (National Taiwan University IRB) and the United States (National Cancer Institute Special Studies IRB). All research was performed in accordance with relevant guidelines and regulations.

We performed WES on 97 families deemed to be most informative for study by virtue of having 3 + NPC cases (N = 35) or 2 affected individuals where at least 2 NPC cases or obligate carriers had biological material available (N = 62). In total, WES was performed on 205 affected individuals, 21 obligate carriers (based on pedigree structure; assuming dominant mode of transmission), and 25 unaffected family members (21 blood relatives and 4 relatives through marriage) (Supplemental Table 1). Relationships between affected family members and between unaffected-affected family members are shown in Supplemental Table 1.

### Whole exome capture and sequencing

Whole-exome capture/sequencing was performed at the NCI Cancer Genomics Research Laboratory (CGR) as previously described^34,35^. Briefly, for each sample, 1.1 μg of genomic DNA extracted from blood was used for exome sequence capture performed with SeqCap EZ Human Exome Library v3.0 (Roche NimbleGen). Pools of captured DNA underwent paired-end sequencing using an Illumina HiSeq according to Illumina-provided protocols for 2 × 100-bp paired-end sequencing. Each exome was sequenced to high depth to achieve a minimum threshold of 80% of coding sequence covered by at least 15 reads, based on the UCSC hg19 ‘known gene’ transcripts. The exome data analyzed in this paper are archived in the CGR exome build Ensemble_New_Annotation dated 2014-01-09.

### Bioinformatics

Details of the bioinformatic pipeline for variant alignment/calling have been published^35,36^. Briefly, sequencing reads were trimmed by Trimmomatic (v0.32)^37^ to remove short read pairs with at least one end <36 bp. Reads were aligned to the hg19 reference genome using Novoalign software (v3.00.05) (http://www.novocraft.com). The alignments were refined by removing duplicate reads in Picard software, MarkDuplicate module (v1.126) (http://picard.sourceforge.net/), short fragments (<200 bp), and sequence pairs not in complementary direction when mapped to reference. Sites with indels were realigned locally by Genome Analysis Toolkit^38^ (GATK v3.1), the RealignerTargetCreator and IndelRealigner modules.

Variant discovery and genotype calling were performed globally among all subjects using UnifiedGenotyper and HaplotypeCaller modules from GATK and the FreeBayes variant caller (v9.9.2). An Ensemble variant calling pipeline (v0.2.2 http://bcb.io/2013/02/06/an-automated-ensemble-method-for-combining-and-evaluating-genomic-variants-from-multiple-callers/) was used to integrate the results from the three callers. Support Vector Machine learning algorithm was used to identify an optimal decision boundary to produce a more balanced decision between false and true positives.

Variants were annotated via a custom CGR in-house script based on public data including Ensembl, refGene, and UCSC KnownGene databases, the dataset from University of Washington’s Exome Sequencing Project (ESP6500) (http://evs.gs.washington.edu/EVS/), dbNSFP^39^: database of human nonsynonymous SNPs and function predictions (https://sites.google.com/site/jpopgen/dbNSFP), Combined Annotation Dependent Depletion (CADD) scores (cadd.gs.washington.edu), the Molecular Signatures Database (MSigDB) (http://www.broadinstitute.org/gsea/msigdb/index.jsp), the National Center for Biotechnology Information (NCBI) Clinically Relevant Sequence Variations (ClinVar) and Single Nucleotide Polymorphism database (dbSNP) databases^40^ build 137, the 1000 Genomes Project^41^, the Exome Aggregation Consortium (ExAC) database (http://exac.broadinstitute.org/), and the Human Gene Mutation Database (HGMD)^42^.

### Filtering criteria and pathway analysis

We applied the following criteria to filter WES variants called in our families. We excluded from consideration (1) variants that had depth of <10 reads or that were in segmentally duplicated regions; (2) variants observed at a frequency >0.5% in the overall or East Asian populations in the 1000 Genomes Project, the NHLBI ESP6500 database, and ExAC database; (3) variants that were observed in >3 families in non-NPC families from our in-house NCI DCEG database of >2,000 sequenced individuals or in >50 individuals in our NPC population (based on CGR exome build Ensemble_New_Annotation dated 2014-01-09). We further applied a dominant model requiring variants to be present in all affected individuals and obligate carriers within a family to reduce the impact of phenocopies or sequencing errors. More specifically, we required that variants be observed (1) in at least 1 family with either three or more NPC cases or two NPC cases and 1 obligate carrier or (2) in at least 1 family for families with two or more NPC cases sequenced that were not siblings or parent-child combinations and for whom at least 1 unaffected individual was sequenced or (3) in at least 2 families for families with two or more NPC cases sequenced that were siblings or parent-child combinations. Within the set of identified variants, we restricted our evaluation to variants most likely to be functional by (1) excluding variants that resulted in synonymous changes (i.e., we retained nonsynonymous, inframe deletions/insertions, frameshift, and nonsense variants and splicing variants), and (2) requiring that the variant was predicted to be deleterious by Combined Annotation Dependent Depletion (CADD) analysis (defined as CADD score of 15 or greater). At least one variant from each of 608 candidate genes were identified using the criteria above. These genes are listed in Supplemental Table 2. To target the most promising findings for further consideration, we performed a literature review and prioritized variants in genes with a reported link to cancer, viral infections or immune-related processes (Table 1).

As a second (unbiased) method for prioritizing candidate genes, we conducted a pathway analysis. Ingenuity Pathway analysis (IPA) was performed (https://www.qiagenbioinformatics.com/products/ingenuity-pathway-analysis/) for the candidate genes in Supplemental Table 2. IPA was performed to examine which pathways were enriched from the candidate genes (Supplemental Table 2). Genes within the pathway that showed the strongest evidence for association with NPC (Notch signaling) were included in our final list of top hits (Table 1). For this set of genes, variants that fully co-segregated with disease in families with at least two NPC cases/obligate carriers sequenced were included in Table 1.

### Technical validation of exome findings

Technical validation of the top candidate variants (listed in Table 1) was performed. Initially, variants were technically validated using Sanger sequencing (n = 6 variants). Subsequently, variants were validated using a targeted, multiplex PCR primer panel comprised of primer pairs designed using the custom Ion AmpliSeq Designer. Sample DNA (30 ng) was amplified using this custom AmpliSeq primer pool, and libraries were prepared following the manufacturer’s Ion AmpliSeq Library Preparation protocol (Life Technologies, Carlsbad, CA, USA). Individual samples were barcoded, pooled, templated, and sequenced on the Ion Torrent PGM Sequencer using the Ion PGM Template OT2 200 and Ion PGM Hi-Q Sequencing 200 kits per manufacturer’s instructions. All variants in Table 1 were confirmed upon technical validation.

### Examination of top variants in publicly available WES datasets of NPC patients

The FASTQ files of 39 early-age onset NPC and 56 NPC germline samples used by two previous studies Lin *et al*.^10^; Dai *et al*.^8^ were downloaded from https://www.ncbi.nlm.nih.gov/sra/?term=SRA291701 and https://www.ncbi.nlm.nih.gov/sra/?term=SRP035573 respectively. Sequence reads in the FASTQ files were trimmed using the Trimmomatic program, which marks all low-quality stretches (average quality score <Q15 in a 4-bp sliding window) and reports the longest high-quality stretch of each read. Only read pairs with both ends no shorter than 36 bp were used. Reads were then aligned to the hg19 reference genome using the Novoalign software (v3.00.05) (http://www.novocraft.com). Duplicate reads due to either optical or PCR artifacts were removed from further analysis using the MarkDuplicates module of the Picard software (v1.126) (http://picard.sourceforge.net/). Additionally, the analysis was restricted to only properly aligned read pairs, in the sense that the two ends of each pair must be mapped to the reference genome in complementary directions and must reflect a reasonable fragment length (300+/−100 bp). These high-quality alignments for each sample were further refined according to a local realignment strategy around known and novel sites of insertion and deletion polymorphisms using the RealignerTargetCreator and IndelRealigner modules from the Genome Analysis Toolkit (GATK v3.8.1). After two batches of BAMs were generated, SNPs and small indels joint calling on the loci presented in Table 1 were performed on the 39 BAMs and 56 BAMs from the two studies using the HaplotypeCaller modules from Genome Analysis Toolkit (GATK v3.8.1).

### Review of previous findings

To evaluate whether previously published and publicly available data on somatic alterations in NPC provide support for the primary findings reported herein, we queried the cBioPortal (cBioPortal.org) and Cosmic (cancer.sanger.ac.uk/cosmic) databases to determine the extent to which alterations in the genes reported herein were noted in past studies. Also, after completion of the current analyses, two WES studies of NPC cases and controls were identified based on published work^8,9^. Although not definitive, *KMT2C* and *MST1R* were proposed as candidate susceptibility genes for NPC. We therefore examined the Taiwanese NPC families with at least two NPC cases/obligate carriers sequenced for rare deleterious variants in these two genes to evaluate evidence for co-segregation of rare variants.

### Magnesium testing

Serum (500 ul) from 13 individuals (6 affected/obligate carriers; 7 unaffected) from two families with a rare NIPAL1 variant were tested for total serum magnesium (NMS Labs, Willow Grove, PA) using an inductively coupled plasma atomic emission spectrometry assay as previously described^43^. To independently examine the possible association between magnesium levels and sporadic NPC, we also evaluated routinely collected clinical magnesium testing results from patients in the ENT clinic at the National Taiwan University Hospital between 07/2010 and 01/2015 and compared the proportion of individuals with total serum magnesium deficiency (defined as <0.7 mmol/L or <0.78 mmol/L depending on instrumentation used) among incident NPC cases (N = 197; 81.7% WHO type I, 16.2% WHO type II, and 2.1% missing histology) and non-NPC controls (N = 237) tested before treatment. Diagnosis of non-NPC controls included diseases of the salivary gland (N = 154), thyroid conditions (N = 31), benign neoplasms of connective and other soft tissue (N = 13), and assorted other conditions (N = 39).

## Supplementary information

Supplemental Tables 1–4
