# Supplementary material for: Whole-Exome Sequencing of Nasopharyngeal Carcinoma Families Reveals Novel Variants Potentially Involved in Nasopharyngeal Carcinoma
Source: Sci Rep. 2019 Jul 9;9:9916. doi: 10.1038/s41598-019-46137-4 (PMC6617453; doi:10.1038/s41598-019-46137-4)
Supplement: Supplementary file 1 — Supplemental Tables 1–4 [file 41598_2019_46137_MOESM1_ESM.pdf]

SUPPLEMENTARY TABLES

Whole-Exome Sequencing of Nasopharyngeal Carcinoma Families Reveals Novel Variants Potentially Involved in Nasopharyngeal Carcinoma

Guoqin Yu[1], Wan-Lun Hsu[2], Anna E. Coghill[3], Kelly J. Yu[3], Cheng-Ping Wang[4], Pei-Jen Lou[4], Zhiwei Liu[3], Kristie Jones[5], Aurelie Vogt[5], Mingyi Wang[5], Sam M. Mbulaiteye[3], Hao-Hui Chen[4], Joseph Boland[5], Meredith Yeager[5], Scott R. Diehl[6], Chien-Jen Chen[2], Allan Hildesheim[3*±], Alisa M. Goldstein[7*±]

Running Title: Whole Exome Sequencing of NPC Families

Supplementary Table 1: Summary of Families Evaluated in the Taiwan NPC Family Study Whole Exome Sequencing Study

Family ID	Number Affected Individuals in Family	Number of Affected Individuals in Family Sequenced	Number of Obligate Carriers in Family Sequenced	Number of Unaffected Individuals in Family Sequenced	Relationships among affected sequenced individuals*	Relationship affected sequenced individuals to sequenced obligate carriers**	Relationship affected sequenced individuals to unaffected sequenced individuals***
NPC_1003	3	2	0	0	Siblings	N/A	N/A
NPC_1004	3	2	0	0	Siblings	N/A	N/A
NPC_1026	10	3	1	1	Other (505, 659, 664)	Son/Sibling/Other	Other
NPC_1028	2	2	0	0	Siblings	N/A	N/A
NPC_1038	2	2	1	1	Other (506, 561)	Daughter/Other	Daughter/Other
NPC_1048	2	2	0	0	Siblings	N/A	N/A
NPC_1061	3	2	1	1	Other (413, 501)	Son/Other	Other
NPC_1062	2	2	1	2	Other (501, 563)	Daughter/Other	Son/Daughter/Other
NPC_1064	2	2	1	0	Siblings	Sons	N/A
NPC_1070	3	2	0	0	Other (501, 561)	N/A	N/A
NPC_1072	3	2	0	0	Siblings	N/A	N/A
NPC_1074	2	2	0	0	Siblings	N/A	N/A
NPC_1076	2	2	0	0	Siblings	N/A	N/A
NPC_1080	2	2	0	0	Siblings	N/A	N/A
NPC_1081	2	2	0	0	Siblings	N/A	N/A
NPC_1082	3	2	0	0	Siblings	N/A	N/A
NPC_1083	2	2	0	0	Siblings	N/A	N/A
NPC_1085	4	2	0	0	Siblings	N/A	N/A
NPC_1086	4	2	0	0	Other (503, 561)	N/A	N/A
NPC_1087	7	3	0	0	Siblings/Other	N/A	N/A
NPC_1094	2	2	0	0	Siblings	N/A	N/A
NPC_1096	3	2	0	0	Other (419, 504)	N/A	N/A
NPC_1108	2	2	0	0	Siblings	N/A	N/A
NPC_2003	2	2	0	0	Siblings	N/A	N/A
NPC_2004	5	3	1	1	Siblings/Other	Sons/Sibling	Sons/Other
NPC_2006	2	2	0	0	Siblings	N/A	N/A
NPC_2007	2	2	1	1	Other (411, 501)	Son/Sibling	Son/Other
NPC_2017	2	2	1	1	Other (412, 503)	Son/Sibling	Son/Other
NPC_2018	3	2	0	0	Siblings	N/A	N/A
NPC_3001	2	2	1	1	Other (411, 501)	Son/Sibling	Son/Other
NPC_3002	2	2	0	0	Siblings	N/A	N/A
NPC_3008	5	3	1	1	Father-Daughter/Other	Son/Other	Daughter/Spouse/Other
NPC_3033	2	2	0	1	Other (414, 502)	N/A	Son/Other
NPC_3034	2	2	0	0	Other (501, 569)	N/A	N/A
NPC_3040	2	2	0	0	Siblings	N/A	N/A
NPC_3049	4	3	0	0	Siblings	N/A	N/A
NPC_3050	3	2	0	0	Siblings	N/A	N/A
NPC_3055	2	2	0	0	Siblings	N/A	N/A
NPC_3056	2	2	1	1	Other (509, 653)	Daughter/Sibling	Daughter/Other
NPC_3062	2	2	0	0	Siblings	N/A	N/A
NPC_3063	2	2	0	0	Siblings	N/A	N/A
NPC_3064	4	2	0	0	Other (507, 658)	N/A	N/A

NPC_3069	3	2	0	0	Siblings	N/A	N/A
NPC_3076	3	3	0	0	Siblings	N/A	N/A
NPC_3083	2	2	0	0	Siblings	N/A	N/A
NPC_3090	3	2	1	0	Other (502, 562)	Daughter/Other	N/A
NPC_3097	2	2	0	0	Siblings	N/A	N/A
NPC_4001	2	2	0	0	Siblings	N/A	N/A
NPC_4004	2	2	1	2	Other (505, 561)	Daughter/Other	Son/Daughter/Other
NPC_4013	3	2	1	0	Other (501, 568)	Son/Other	N/A
NPC_4031	3	3	0	0	Siblings	N/A	N/A
NPC_4037	2	2	0	2	Other (501, 561)	N/A	Son/Daughter/Other
NPC_4047	4	2	0	1	Other (416, 501)	N/A	Son/Other
NPC_4058	2	2	0	0	Siblings	N/A	N/A
NPC_4060	2	2	0	0	Siblings	N/A	N/A
NPC_4061	2	2	0	0	Siblings	N/A	N/A
NPC_4063	2	2	0	0	Siblings	N/A	N/A
NPC_4077	3	2	0	0	Siblings	N/A	N/A
NPC_4082	7	4	0	0	Mother-Son/Siblings/Other	N/A	N/A
NPC_4084	3	2	0	0	Siblings	N/A	N/A
NPC_4097	2	2	0	0	Siblings	N/A	N/A
NPC_4099	4	2	0	0	Other (504, 561)	N/A	N/A
NPC_4105	2	2	0	0	Siblings	N/A	N/A
NPC_4113	2	2	0	0	Other (412, 501)	N/A	N/A
NPC_5001	2	2	0	0	Siblings	N/A	N/A
NPC_5003	2	2	0	0	Siblings	N/A	N/A
NPC_5004	2	2	0	1	Other (503, 563)	N/A	Son/Other
NPC_5006	2	2	1	2	Other (304, 501)	Mother/Son	Son/Spouse/Other
NPC_5012	3	2	0	0	Siblings	N/A	N/A
NPC_5013	4	2	0	1	Mother-Son	N/A	Son/Spouse
NPC_5019	4	2	0	0	Other (601, 654)	N/A	N/A
NPC_5036	3	2	0	0	Father-Daughter	N/A	N/A
NPC_5040	4	3	0	0	Siblings	N/A	N/A
NPC_5041	5	2	1	0	Other (506, 575)	Son/Other	N/A
NPC_5051	2	2	0	0	Siblings	N/A	N/A
NPC_5054	2	2	1	2	Other (501, 562)	Son/Other	Son/Other
NPC_5065	3	2	0	0	Siblings	N/A	N/A
NPC_5076	3	2	1	1	Siblings	Sons	Sons
NPC_5078	2	2	0	0	Siblings	N/A	N/A
NPC_5087	5	2	0	0	Siblings	N/A	N/A
NPC_5088	3	2	0	0	Siblings	N/A	N/A
NPC_5089	2	2	0	0	Siblings	N/A	N/A
NPC_5092	2	2	0	0	Siblings	N/A	N/A
NPC_5094	2	2	0	0	Siblings	N/A	N/A
NPC_5096	2	2	0	0	Other (507, 564)	N/A	N/A
NPC_5098	3	2	1	0	Other (503, 566)	Son/Other	N/A
NPC_5100	2	2	0	0	Siblings	N/A	N/A
NPC_5102	2	2	0	0	Siblings	N/A	N/A
NPC_5105	2	2	0	0	Other (501, 562)	N/A	N/A
NPC_5106	4	3	0	0	Siblings	N/A	N/A

NPC_6002	2	2	0	0	Father-Daughter	N/A	N/A
NPC_6012	2	2	0	0	Siblings	N/A	N/A
NPC_6014	3	2	1	0	Other (414, 501)	Daughter/Sibling	N/A
NPC_6015	2	2	1	1	Other (503, 563)	Son/Other	Son/Other
NPC_6016	2	2	0	0	Siblings	N/A	N/A
NPC_6036	4	2	0	0	Other (602, 651)	N/A	N/A
NPC_6040	3	2	0	0	Siblings	N/A	N/A

* Relationship of "Other" includes second degree (N=17), third degree (N=23), and >third degree (N=1) relatives.

** Relationship of "Other" includes second degree relatives (N=11).

** Relationship of "Other" includes second degree relatives (N=3) and relatives by marriage (N=21).

Supplementary Table 2: List of Candidate Genes Identified in the Taiwan NPC Family Whole Exome Sequencing Study

Chromosome	Ensemble_Gene_ID	Gene
chr1	ENSG00000130762	ARHGEF16
chr1	ENSG00000162592	CCDC27
chr1	ENSG00000131697	NPHP4
chr1	ENSG00000171680	PLEKHG5
chr1	ENSG00000074800	ENO1
chr1	ENSG00000180758	GPR157
chr1	ENSG00000171824	EXOSC10
chr1	ENSG00000116661	FBXO2
chr1	ENSG00000083444	PLOD1
chr1	ENSG00000162494	LRRC38
chr1	ENSG00000116138	DNAJC16
chr1	ENSG00000142623	PADI1
chr1	ENSG00000142619	PADI3
chr1	ENSG00000074964	ARHGEF10L
chr1	ENSG00000127463	EMC1
chr1	ENSG00000158816	VWA5B1
chr1	ENSG00000090432	MUL1
chr1	ENSG00000075151	EIF4G3
chr1	ENSG00000158008	EXTL1
chr1	ENSG00000142748	FCN3
chr1	ENSG00000084652	TXLNA
chr1	ENSG00000162520	SYNC
chr1	ENSG00000092853	CLSPN
chr1	ENSG00000090621	PABPC4
chr1	ENSG00000049089	COL9A2
chr1	ENSG00000117016	RIMS3
chr1	ENSG00000127129	EDN2
chr1	ENSG00000186409	CCDC30
chr1	ENSG00000117425	PTCH2
chr1	ENSG00000132763	MMACHC
chr1	ENSG00000142961	MOB3C
chr1	ENSG00000186564	FOXD2
chr1	ENSG00000162390	ACOT11
chr1	ENSG00000162407	PPAP2B
chr1	ENSG00000134709	HOOK1
chr1	ENSG00000118473	SGIP1
chr1	ENSG00000081985	IL12RB2
chr1	ENSG00000162618	ELTD1
chr1	ENSG00000097033	SH3GLB1
chr1	ENSG00000122435	TRMT13
chr1	ENSG00000116337	AMPD2
chr1	ENSG00000121933	ADORA3
chr1	ENSG00000118655	DCLRE1B

chr1	ENSG00000163393	SLC22A15
chr1	ENSG00000159409	CELF3
chr1	ENSG00000116539	ASH1L
chr1	ENSG00000163472	TMEM79
chr1	ENSG00000132677	RHBG
chr1	ENSG00000152092	ASTN1
chr1	ENSG00000075391	RASAL2
chr1	ENSG00000058085	LAMC2
chr1	ENSG00000116679	IVNS1ABP
chr1	ENSG00000177888	ZBTB41
chr1	ENSG00000116852	KIF21B
chr1	ENSG00000117139	KDM5B
chr1	ENSG00000143847	PPFIA4
chr1	ENSG00000184144	CNTN2
chr1	ENSG00000143486	EIF2D
chr1	ENSG00000117597	DIEXF
chr1	ENSG00000162909	CAPN2
chr1	ENSG00000143801	PSEN2
chr1	ENSG00000135763	URB2
chr1	ENSG00000135773	CAPN9
chr1	ENSG00000054267	ARID4B
chr1	ENSG00000153187	HNRNPU
chr1	ENSG00000162849	KIF26B
chr2	ENSG00000134324	LPIN1
chr2	ENSG00000214842	RAD51AP2
chr2	ENSG00000118965	WDR35
chr2	ENSG00000119771	KLHL29
chr2	ENSG00000163811	WDR43
chr2	ENSG00000158125	XDH
chr2	ENSG00000018699	TTC27
chr2	ENSG00000115825	PRKD3
chr2	ENSG00000119787	ATL2
chr2	ENSG00000138095	LRPPRC
chr2	ENSG00000119878	CRIPT
chr2	ENSG00000119737	GPR75
chr2	ENSG00000177994	C2orf73
chr2	ENSG00000055813	CCDC85A
chr2	ENSG00000162928	PEX13
chr2	ENSG00000115956	PLEK
chr2	ENSG00000124357	NAGK
chr2	ENSG00000239779	WBP1
chr2	ENSG00000159374	M1AP
chr2	ENSG00000135622	SEMA4F
chr2	ENSG00000115364	MRPL19
chr2	ENSG00000115525	ST3GAL5
chr2	ENSG00000144026	ZNF514
chr2	ENSG00000168758	SEMA4C
chr2	ENSG00000144218	AFF3
chr2	ENSG00000125637	PSD4

chr2	ENSG00000125618	PAX8
chr2	ENSG00000125633	CCDC93
chr2	ENSG00000115107	STEAP3
chr2	ENSG00000163075	PCDP1
chr2	ENSG00000072163	LIMS2
chr2	ENSG00000144230	GPR17
chr2	ENSG00000115221	ITGB6
chr2	ENSG00000144290	SLC4A10
chr2	ENSG00000144285	SCN1A
chr2	ENSG00000115806	GORASP2
chr2	ENSG00000115840	SLC25A12
chr2	ENSG00000152256	PDK1
chr2	ENSG00000128654	MTX2
chr2	ENSG00000116044	NFE2L2
chr2	ENSG00000173166	RAPH1
chr2	ENSG00000127837	AAMP
chr2	ENSG00000135912	TTLL4
chr2	ENSG00000115657	ABCB6
chr2	ENSG00000198925	ATG9A
chr2	ENSG00000085449	WDFY1
chr2	ENSG00000135900	MRPL44
chr2	ENSG00000135899	SP110
chr2	ENSG00000067066	SP100
chr2	ENSG00000085978	ATG16L1
chr2	ENSG00000132321	IQCA1
chr2	ENSG00000142327	RNPEPL1
chr2	ENSG00000006607	FARP2
chr3	ENSG00000168137	SETD5
chr3	ENSG00000157014	TATDN2
chr3	ENSG00000177463	NR2C2
chr3	ENSG00000131386	GALNT15
chr3	ENSG00000183960	KCNH8
chr3	ENSG00000076242	MLH1
chr3	ENSG00000144677	CTDSPL
chr3	ENSG00000168026	TTC21A
chr3	ENSG00000144659	SLC25A38
chr3	ENSG00000160746	ANO10
chr3	ENSG00000114648	KLHL18
chr3	ENSG00000114270	COL7A1
chr3	ENSG00000233276	GPX1
chr3	ENSG00000145022	TCTA
chr3	ENSG00000164068	RNF123
chr3	ENSG00000176020	AMIGO3
chr3	ENSG00000001617	SEMA3F
chr3	ENSG00000114378	HYAL1
chr3	ENSG00000041880	PARP3
chr3	ENSG00000164087	POC1A
chr3	ENSG00000168237	GLYCTK
chr3	ENSG00000163939	PBRM1

chr3	ENSG00000055957	ITIH1
chr3	ENSG00000157388	CACNA1D
chr3	ENSG00000144724	PTPRG
chr3	ENSG00000163638	ADAMTS9
chr3	ENSG00000172340	SUCLG2
chr3	ENSG00000185008	ROBO2
chr3	ENSG00000169855	ROBO1
chr3	ENSG00000044524	EPHA3
chr3	ENSG00000198919	DZIP3
chr3	ENSG00000144824	PHLDB2
chr3	ENSG00000163617	KIAA1407
chr3	ENSG00000051341	POLQ
chr3	ENSG00000114030	KPNA1
chr3	ENSG00000138246	DNAJC13
chr3	ENSG00000114019	AMOTL2
chr3	ENSG00000090402	SI
chr3	ENSG00000171109	MFN1
chr3	ENSG00000114867	EIF4G1
chr3	ENSG00000113889	KNG1
chr3	ENSG00000127241	MASP1
chr4	ENSG00000174227	PIGG
chr4	ENSG00000087274	ADD1
chr4	ENSG00000188981	MSANTD1
chr4	ENSG00000087008	ACOX3
chr4	ENSG00000155275	TRMT44
chr4	ENSG00000178163	ZNF518B
chr4	ENSG00000174130	TLR6
chr4	ENSG00000124406	ATP8A1
chr4	ENSG00000151806	GUF1
chr4	ENSG00000163293	NIPAL1
chr4	ENSG00000075539	FRYL
chr4	ENSG00000134852	CLOCK
chr4	ENSG00000090989	EXOC1
chr4	ENSG00000156140	ADAMTS3
chr4	ENSG00000079557	AFM
chr4	ENSG00000029559	IBSP
chr4	ENSG00000118762	PKD2
chr4	ENSG00000163644	PPM1K
chr4	ENSG00000184305	CCSER1
chr4	ENSG00000198099	ADH4
chr4	ENSG00000109323	MANBA
chr4	ENSG00000164038	SLC9B2
chr4	ENSG00000138795	LEF1
chr4	ENSG00000145390	USP53
chr4	ENSG00000085871	MGST2
chr4	ENSG00000183090	FREM3
chr4	ENSG00000170390	DCLK2
chr4	ENSG00000198589	LRBA
chr4	ENSG00000109686	SH3D19

chr4	ENSG00000109762	SNX25
chr4	ENSG00000154556	SORBS2
chr5	ENSG00000049656	CLPTM1L
chr5	ENSG00000164151	KIAA0947
chr5	ENSG00000037474	NSUN2
chr5	ENSG00000197603	C5orf42
chr5	ENSG00000172239	PAIP1
chr5	ENSG00000177058	SLC38A9
chr5	ENSG00000152670	DDX4
chr5	ENSG00000155545	MIER3
chr5	ENSG00000164347	GFM2
chr5	ENSG00000145685	LHFPL2
chr5	ENSG00000177034	MTX3
chr5	ENSG00000113318	MSH3
chr5	ENSG00000131730	CKMT2
chr5	ENSG00000164292	RHOBTB3
chr5	ENSG00000174132	FAM174A
chr5	ENSG00000112874	NUDT12
chr5	ENSG00000138829	FBN2
chr5	ENSG00000112984	KIF20A
chr5	ENSG00000094880	CDC23
chr5	ENSG00000120733	KDM3B
chr5	ENSG00000120725	SIL1
chr5	ENSG00000184584	TMEM173
chr5	ENSG00000170445	HARS
chr5	ENSG00000239389	PCDHA13
chr5	ENSG00000253537	PCDHGA7
chr5	ENSG00000169302	STK32A
chr5	ENSG00000132915	PDE6A
chr5	ENSG00000113716	HMGXB3
chr5	ENSG00000145901	TNIP1
chr5	ENSG00000197043	ANXA6
chr5	ENSG00000186335	SLC36A2
chr5	ENSG00000145934	TENM2
chr5	ENSG00000131187	F12
chr5	ENSG00000161021	MAML1
chr5	ENSG00000037280	FLT4
chr6	ENSG00000112685	EXOC2
chr6	ENSG00000047579	DTNBP1
chr6	ENSG00000172197	MBOAT1
chr6	ENSG00000204560	DHX16
chr6	ENSG00000204256	BRD2
chr6	ENSG00000223501	VPS52
chr6	ENSG00000146197	SCUBE3
chr6	ENSG00000171611	PTCRA
chr6	ENSG00000182793	GSTA5
chr6	ENSG00000124743	KLHL31
chr6	ENSG00000124749	COL21A1
chr6	ENSG00000112232	KHDRBS2

chr6	ENSG00000112280	COL9A1
chr6	ENSG00000112706	IMPG1
chr6	ENSG00000188994	ZNF292
chr6	ENSG00000123552	USP45
chr6	ENSG00000152894	PTPRK
chr6	ENSG00000135541	AHI1
chr6	ENSG00000135540	NHSL1
chr6	ENSG00000001036	FUCA2
chr7	ENSG00000164828	SUN1
chr7	ENSG00000106003	LFNG
chr7	ENSG00000005108	THSD7A
chr7	ENSG00000156928	MALSU1
chr7	ENSG00000164548	TRA2A
chr7	ENSG00000086289	EPDR1
chr7	ENSG00000127947	PTPN12
chr7	ENSG00000205413	SAMD9
chr7	ENSG00000004799	PDK4
chr7	ENSG00000197093	GAL3ST4
chr7	ENSG00000196411	EPHB4
chr7	ENSG00000146828	SLC12A9
chr7	ENSG00000106367	AP1S1
chr7	ENSG00000167011	NAT16
chr7	ENSG00000105821	DNAJC2
chr7	ENSG00000105976	MET
chr7	ENSG00000001626	CFTR
chr7	ENSG00000170807	LMOD2
chr7	ENSG00000106554	CHCHD3
chr7	ENSG00000122786	CALD1
chr7	ENSG00000105948	TTC26
chr7	ENSG00000174469	CNTNAP2
chr7	ENSG00000106479	ZNF862
chr7	ENSG00000055609	MLL3
chr8	ENSG00000168077	SCARA3
chr8	ENSG00000189233	NUGGC
chr8	ENSG00000172728	FUT10
chr8	ENSG00000147471	PROSC
chr8	ENSG00000104365	IKBKB
chr8	ENSG00000147434	CHRNA6
chr8	ENSG00000253729	PRKDC
chr8	ENSG00000147488	ST18
chr8	ENSG00000047249	ATP6V1H
chr8	ENSG00000104237	RP1
chr8	ENSG00000185728	YTHDF3
chr8	ENSG00000147573	TRIM55
chr8	ENSG00000170289	CNGB3
chr8	ENSG00000164796	CSMD3
chr8	ENSG00000136999	NOV
chr8	ENSG00000185189	NRBP2
chr8	ENSG00000187954	CYHR1

chr8	ENSG00000167702	KIFC2
chr9	ENSG00000107077	KDM4C
chr9	ENSG00000107186	MPDZ
chr9	ENSG00000155875	FAM154A
chr9	ENSG00000137106	GRHPR
chr9	ENSG00000070601	FRMPD1
chr9	ENSG00000196781	TLE1
chr9	ENSG00000130957	FBP2
chr9	ENSG00000095383	TBC1D2
chr9	ENSG00000119508	NR4A3
chr9	ENSG00000119457	SLC46A2
chr9	ENSG00000119431	HDHD3
chr9	ENSG00000182752	PAPPA
chr9	ENSG00000119402	FBXW2
chr9	ENSG00000185585	OLFML2A
chr9	ENSG00000136830	FAM129B
chr9	ENSG00000167118	URM1
chr9	ENSG00000197694	SPTAN1
chr9	ENSG00000123454	DBH
chr9	ENSG00000148400	NOTCH1
chr9	ENSG00000176058	TPRN
chr9	ENSG00000197768	C9orf173
chr9	ENSG00000188833	ENTPD8
chr9	ENSG00000130653	PNPLA7
chr9	ENSG00000148399	WDR85
chr10	ENSG00000165983	PTER
chr10	ENSG00000107863	ARHGAP21
chr10	ENSG00000095739	BAMBI
chr10	ENSG00000183621	ZNF438
chr10	ENSG00000099250	NRP1
chr10	ENSG00000148498	PARD3
chr10	ENSG00000107618	RBP3
chr10	ENSG00000070748	CHAT
chr10	ENSG00000156502	SUPV3L1
chr10	ENSG00000107731	UNC5B
chr10	ENSG00000233745	ZNF503
chr10	ENSG00000151208	DLG5
chr10	ENSG00000185303	SFTPA2
chr10	ENSG00000173239	LIPM
chr10	ENSG00000185745	IFIT1
chr10	ENSG00000155287	SLC25A28
chr10	ENSG00000165868	HSPA12A
chr10	ENSG00000187164	KIAA1598
chr10	ENSG00000165650	PDZD8
chr10	ENSG00000107651	SEC23IP
chr10	ENSG00000120008	WDR11
chr10	ENSG00000196177	ACADSB
chr10	ENSG00000065154	OAT

chr10	ENSG00000089876	DHX32
chr10	ENSG00000176769	TCERG1L
chr10	ENSG00000130640	TUBGCP2
chr11	ENSG00000110619	CARS
chr11	ENSG00000132275	RRP8
chr11	ENSG00000188487	INSC
chr11	ENSG00000129167	TPH1
chr11	ENSG00000148935	GAS2
chr11	ENSG00000151348	EXT2
chr11	ENSG00000175274	TP53I11
chr11	ENSG00000110514	MADD
chr11	ENSG00000134571	MYBPC3
chr11	ENSG00000165923	AGBL2
chr11	ENSG00000255223	OR5M11
chr11	ENSG00000149476	DAK
chr11	ENSG00000162144	CYB561A3
chr11	ENSG00000149489	ROM1
chr11	ENSG00000197901	SLC22A6
chr11	ENSG00000168071	CCDC88B
chr11	ENSG00000175634	RPS6KB2
chr11	ENSG00000186635	ARAP1
chr11	ENSG00000175567	UCP2
chr11	ENSG00000054938	CHRDL2
chr11	ENSG00000166435	XRRA1
chr11	ENSG00000158555	GDPD5
chr11	ENSG00000166391	MOGAT2
chr11	ENSG00000149256	TENM4
chr11	ENSG00000137494	ANKRD42
chr11	ENSG00000137500	CCDC90B
chr11	ENSG00000077616	NAALAD2
chr11	ENSG00000150768	DLAT
chr11	ENSG00000160584	SIK3
chr11	ENSG00000118058	MLL
chr11	ENSG00000019144	PHLDB1
chr11	ENSG00000160695	VPS11
chr11	ENSG00000109944	C11orf63
chr11	ENSG00000151503	NCAPD3
chr12	ENSG00000073614	KDM5A
chr12	ENSG00000002016	RAD52
chr12	ENSG00000206044	LOC100507424
chr12	ENSG00000111319	SCNN1A
chr12	ENSG00000111700	SLCO1B3
chr12	ENSG00000134538	SLCO1B1
chr12	ENSG00000111711	GOLT1B
chr12	ENSG00000139160	METTL20
chr12	ENSG00000139117	CPNE8
chr12	ENSG00000151233	GXYLT1
chr12	ENSG00000079337	RAPGEF3
chr12	ENSG00000186049	KRT73

chr12	ENSG00000094914	AAAS
chr12	ENSG00000123364	HOXC13
chr12	ENSG00000205327	OR6C68
chr12	ENSG00000135424	ITGA7
chr12	ENSG00000257727	CNPY2
chr12	ENSG00000166986	MARS
chr12	ENSG00000135407	AVIL
chr12	ENSG00000067798	NAV3
chr12	ENSG00000198707	CEP290
chr12	ENSG00000139323	POC1B
chr12	ENSG00000120868	APAF1
chr12	ENSG00000185480	PARPBP
chr12	ENSG00000111696	NT5DC3
chr12	ENSG00000198855	FICD
chr12	ENSG00000174527	MYO1H
chr12	ENSG00000139436	GIT2
chr12	ENSG00000111231	GPN3
chr12	ENSG00000204842	ATXN2
chr12	ENSG00000111275	ALDH2
chr12	ENSG00000173064	HECTD4
chr12	ENSG00000089009	RPL6
chr12	ENSG00000089250	NOS1
chr12	ENSG00000122966	CIT
chr12	ENSG00000184209	SNRNP35
chr12	ENSG00000188026	RILPL1
chr12	ENSG00000111364	DDX55
chr12	ENSG00000060709	RIMBP2
chr12	ENSG00000247077	PGAM5
chr13	ENSG00000133110	POSTN
chr13	ENSG00000183722	LHFP
chr13	ENSG00000133103	COG6
chr13	ENSG00000102531	FNDC3A
chr14	ENSG00000165762	OR4K2
chr14	ENSG00000221977	OR4E2
chr14	ENSG00000179933	C14orf119
chr14	ENSG00000166091	CMTM5
chr14	ENSG00000100867	DHRS2
chr14	ENSG00000186648	LRRC16B
chr14	ENSG00000196497	IPO4
chr14	ENSG00000100445	SDR39U1
chr14	ENSG00000129514	FOXA1
chr14	ENSG00000262355	SAMD4A
chr14	ENSG00000151812	SLC35F4
chr14	ENSG00000126822	PLEKHG3
chr14	ENSG00000171723	GPHN
chr14	ENSG00000198732	SMOC1
chr14	ENSG00000119599	DCAF4
chr14	ENSG00000119661	DNAL1
chr14	ENSG00000119685	TTLL5

chr14	ENSG00000066455	GOLGA5
chr14	ENSG00000197249	SERPINA1
chr14	ENSG00000186910	SERPINA11
chr14	ENSG00000165953	SERPINA12
chr14	ENSG00000066739	ATG2B
chr14	ENSG00000182218	HHIPL1
chr14	ENSG00000022976	ZNF839
chr15	ENSG00000134160	TRPM1
chr15	ENSG00000198838	RYR3
chr15	ENSG00000159248	GJD2
chr15	ENSG00000140320	BAHD1
chr15	ENSG00000128908	INO80
chr15	ENSG00000159337	PLA2G4D
chr15	ENSG00000092529	CAPN3
chr15	ENSG00000242028	HYPK
chr15	ENSG00000104133	SPG11
chr15	ENSG00000140279	DUOX2
chr15	ENSG00000138592	USP8
chr15	ENSG00000069966	GNB5
chr15	ENSG00000171956	FOXB1
chr15	ENSG00000103591	AAGAB
chr15	ENSG00000188779	SKOR1
chr15	ENSG00000066933	MYO9A
chr15	ENSG00000103740	ACSBG1
chr15	ENSG00000058335	RASGRF1
chr15	ENSG00000103723	AP3B2
chr15	ENSG00000064726	BTBD1
chr15	ENSG00000213471	TTLL13
chr15	ENSG00000140575	IQGAP1
chr16	ENSG00000103126	AXIN1
chr16	ENSG00000103197	TSC2
chr16	ENSG00000059122	FLYWCH1
chr16	ENSG00000140993	TIGD7
chr16	ENSG00000126602	TRAP1
chr16	ENSG00000175595	ERCC4
chr16	ENSG00000103319	EEF2K
chr16	ENSG00000166851	PLK1
chr16	ENSG00000134398	ERN2
chr16	ENSG00000177200	CHD9
chr16	ENSG00000087245	MMP2
chr16	ENSG00000125107	CNOT1
chr16	ENSG00000102890	ELMO3
chr16	ENSG00000103047	TANGO6
chr16	ENSG00000064270	ATP2C2
chr16	ENSG00000153786	ZDHHC7
chr16	ENSG00000131149	GSE1
chr16	ENSG00000205022	PABPN1L
chr17	ENSG00000159842	ABR
chr17	ENSG00000132361	CLUH

chr17	ENSG00000213977	TAX1BP3
chr17	ENSG00000141503	MINK1
chr17	ENSG00000177294	FBXO39
chr17	ENSG00000215041	NEURL4
chr17	ENSG00000182224	CYB5D1
chr17	ENSG00000133026	MYH10
chr17	ENSG00000133020	MYH8
chr17	ENSG00000109061	MYH1
chr17	ENSG00000076382	SPAG5
chr17	ENSG00000132589	FLOT2
chr17	ENSG00000196535	MYO18A
chr17	ENSG00000141161	UNC45B
chr17	ENSG00000006114	SYNRG
chr17	ENSG00000141738	GRB7
chr17	ENSG00000108349	CASC3
chr17	ENSG00000173908	KRT28
chr17	ENSG00000187242	KRT12
chr17	ENSG00000212724	KRTAP2-3
chr17	ENSG00000173801	JUP
chr17	ENSG00000131473	ACLY
chr17	ENSG00000108797	CNTNAP1
chr17	ENSG00000004939	SLC4A1
chr17	ENSG00000013306	SLC25A39
chr17	ENSG00000198933	TBKBP1
chr17	ENSG00000141295	SCRN2
chr17	ENSG00000159184	HOXB13
chr17	ENSG00000159210	SNF8
chr17	ENSG00000108821	COL1A1
chr17	ENSG00000136449	MYCBPAP
chr17	ENSG00000006283	CACNA1G
chr17	ENSG00000153944	MSI2
chr17	ENSG00000121053	EPX
chr17	ENSG00000173826	KCNH6
chr17	ENSG00000177303	CASKIN2
chr17	ENSG00000188126	MYO15B
chr17	ENSG00000132470	ITGB4
chr17	ENSG00000132478	UNK
chr17	ENSG00000250506	CDK3
chr17	ENSG00000173894	CBX2
chr17	ENSG00000169710	FASN
chr17	ENSG00000141568	FOXK2
chr18	ENSG00000101557	USP14
chr18	ENSG00000154845	PPP4R1
chr18	ENSG00000185231	MC2R
chr18	ENSG00000101752	MIB1
chr18	ENSG00000170558	CDH2
chr18	ENSG00000134765	DSC1
chr18	ENSG00000167306	MYO5B
chr18	ENSG00000049759	NEDD4L

chr18	ENSG00000176225	RTTN
chr18	ENSG00000215421	ZNF407
chr18	ENSG00000166377	ATP9B
chr19	ENSG00000129946	SHC2
chr19	ENSG00000104885	DOT1L
chr19	ENSG00000127666	TICAM1
chr19	ENSG00000127663	KDM4B
chr19	ENSG00000212123	PRR22
chr19	ENSG00000167772	ANGPTL4
chr19	ENSG00000142347	MYO1F
chr19	ENSG00000167785	ZNF558
chr19	ENSG00000244165	P2RY11
chr19	ENSG00000130816	DNMT1
chr19	ENSG00000105397	TYK2
chr19	ENSG00000129354	AP1M2
chr19	ENSG00000079805	DNM2
chr19	ENSG00000104915	STX10
chr19	ENSG00000105135	ILVBL
chr19	ENSG00000105131	EPHX3
chr19	ENSG00000072954	TMEM38A
chr19	ENSG00000254901	MEF2BNB
chr19	ENSG00000064490	RFXANK
chr19	ENSG00000121297	TSHZ3
chr19	ENSG00000105220	GPI
chr19	ENSG00000105270	CLIP3
chr19	ENSG00000090932	DLL3
chr19	ENSG00000176401	EID2B
chr19	ENSG00000105202	FBL
chr19	ENSG00000105323	HNRNPUL1
chr19	ENSG00000105429	MEGF8
chr19	ENSG00000007255	TRAPPC6A
chr19	ENSG00000104941	RSPH6A
chr19	ENSG00000174951	FUT1
chr19	ENSG00000126453	BCL2L12
chr19	ENSG00000086967	MYBPC2
chr19	ENSG00000125503	PPP1R12C
chr19	ENSG00000204519	ZNF551
chr20	ENSG00000149488	TMC2
chr20	ENSG00000215251	FASTKD5
chr20	ENSG00000125885	MCM8
chr20	ENSG00000149346	SLX4IP
chr20	ENSG00000125844	RRBP1
chr20	ENSG00000188559	RALGAPA2
chr20	ENSG00000100997	ABHD12
chr20	ENSG00000167104	BPIFB6
chr20	ENSG00000088367	EPB41L1
chr20	ENSG00000080839	RBL1
chr20	ENSG00000196090	PTPRT
chr20	ENSG00000132824	SERINC3

chr21	ENSG00000180530	NRIP1
chr21	ENSG00000154646	TMPRSS15
chr21	ENSG00000198862	LTN1
chr21	ENSG00000156284	CLDN8
chr21	ENSG00000142197	DOPEY2
chr21	ENSG00000185658	BRWD1
chr21	ENSG00000160214	RRP1
chr22	ENSG00000070371	CLTCL1
chr22	ENSG00000178803	ADORA2A-AS1
chr22	ENSG00000100154	TTC28
chr22	ENSG00000186998	EMID1
chr22	ENSG00000184792	OSBP2
chr22	ENSG00000184708	EIF4ENIF1
chr22	ENSG00000100060	MFNG
chr22	ENSG00000100065	CARD10
chr22	ENSG00000256872	NOL12
chr22	ENSG00000128298	BAIAP2L2
chr22	ENSG00000100242	SUN2
chr22	ENSG00000100316	RPL3
chr22	ENSG00000100354	TNRC6B
chr22	ENSG00000172346	CSDC2
chr22	ENSG00000167077	MEI1
chr22	ENSG00000100266	PACSIN2
chr22	ENSG00000100300	TSPO
chr22	ENSG00000186654	PRR5
chr22	ENSG00000186951	PPARA
chr22	ENSG00000075234	TTC38

Supplemental Table 3: List of All Rare, Nonsynonymous/Loss-of-Function Variants Observed for the Candidate Genes from Table 1

Gene Name	Chromosome	Position	Rs ID	Ref	Var	Change observed	cytoband	(No. NPC cases/obligate carriers (OC) with variant)/(total # NPC cases + OC sequenced)	ID of family(ies) in which variant observed	KG_AF	KG_EAS_AF	ESP_AA_AF	ESP_EA_AF	ExAC_ALL	ExAC_EAS
BCL2L12	chr19	50169135	rs771347725	C	T	nonsynonymous_SNV	19q13.33	2/2; 2/2	6016; 4061	0	0	0	0	1.67E-05	2.00E-04
BCL2L12	chr19	50172146	rs201397773	C	T	nonsynonymous_SNV	19q13.33	1/1; 1/3; 1/2	4023; 5002; 5019	5.99E-04	0.003	0	0	2.00E-04	0.0023
BRD2	chr6	32945580	.	A	T	nonsynonymous_SNV	6p21.32	1/3	4004	0	0	0	0	0	0
BRD2	chr6	32948153	rs778675391	A	T	nonsynonymous_SNV	6p21.32	2/2; 1/2; 2/2; 1/1; 1/1; 2/2	1003; 1081; 3002; 3079; 4045; 4105	0	0	0	0	3.00E-04	0.0034
CLPTM1L	chr5	1320735	.	G	A	nonsynonymous_SNV	5p15.33	3/3	6014	0	0	0	0	0	0
DLL3	chr19	39991273	rs777813964	A	G	nonsynonymous_SNV	19q13.2	3/3	4013	0	0	0	0	3.30E-05	5.00E-04
DLL3	chr19	39994903	rs368487646	C	T	nonsynonymous_SNV	19q13.2	1/2	1082	0	0	0	1.16E-04	0	0
HNRNPU	chr1	245021539	rs760669739	T	C	nonsynonymous_SNV	1q44	2/2; 2/2; 2/2	3050; 4060; 5078	0	0	0	0	2.00E-04	0.0024
HNRNPU	chr1	245027475	rs775075938	CTCG	C	nonframeshift_deletion	1q44	2/2	3063	0	0	0	0	1.37E-05	0
ITGB6	chr2	160993948	rs188076755	C	T	nonsynonymous_SNV	2q24.2	2/2	5013	0	0	0	0	6.10E-05	5.00E-04
ITGB6	chr2	160994013	.	T	C	nonsynonymous_SNV	2q24.2	1/2	4097	0	0	0	0	0	0
ITGB6	chr2	160994068	rs199564092	C	T	nonsynonymous_SNV	2q24.2	2/3	5106	2.00E-04	0	0	1.16E-04	3.00E-04	5.00E-04
ITGB6	chr2	160994128	.	C	T	nonsynonymous_SNV	2q24.2	1/1	5009	0	0	0	0	0	0
ITGB6	chr2	161051893	rs764843609	C	T	nonsynonymous_SNV	2q24.2	2/2	1074	0	0	0	0	3.30E-05	5.00E-04
MLH1	chr3	37050378	.	T	G	nonsynonymous_SNV	3p22.2	1/2	1083	0	0	0	0	0	0
MLH1	chr3	37053562	rs4986984	C	T	nonsynonymous_SNV	3p22.2	1/2; 2/2	2018; 4060	5.99E-04	0.002	0	0	3.00E-04	0.0039
MLH1	chr3	37067192	rs201673334	C	T	nonsynonymous_SNV	3p22.2	3/3	3001	2.00E-04	0.001	0	0	2.49E-05	3.00E-04
MLH1	chr3	37070352	rs63750226	C	G	nonsynonymous_SNV	3p22.2	1/1	1097	0	0	0	0	4.94E-05	3.00E-04
MLH1	chr3	37090015	rs63751270	A	G	nonsynonymous_SNV	3p22.2	2/4	4082	0	0	0	0	0	0
NEDD4L	chr18	55998058	rs372196719	G	A	nonsynonymous_SNV	18q21.31	1/1	1073	0	0	2.69E-04	1.22E-04	1.66E-05	0
NEDD4L	chr18	56002735	rs373080858	G	A	nonsynonymous_SNV	18q21.31	2/2	3033	0	0	3.19E-04	0	4.16E-05	3.00E-04
NEDD4L	chr18	56016815	.	C	T	nonsynonymous_SNV	18q21.31	1/2	5100	0	0	0	0	0	0
NIPAL1	chr4	48027270	rs556842110	T	C	nonsynonymous_SNV	4p12	2/2	3064	2.00E-04	0.001	0	0	9.06E-05	0.0013
NIPAL1	chr4	48032135	rs563722989	A	G	splicing	4p12	4/4	4082	2.00E-04	0.001	0	0	2.51E-05	3.00E-04
NOTCH1	chr9	139390950	.	G	A	nonsynonymous_SNV	9q34.3	1/2	3034	0	0	0	0	0	0
NOTCH1	chr9	139391476	rs747504082	C	T	nonsynonymous_SNV	9q34.3	1/3	5106	0	0	0	0	6.16E-05	5.00E-04
NOTCH1	chr9	139396278	rs376689092	G	A	nonsynonymous_SNV	9q34.3	1/1	1020	0	0	2.38E-04	0	8.52E-06	0
NOTCH1	chr9	139396363	rs778371465	G	C	nonsynonymous_SNV	9q34.3	1/3	6015	0	0	0	0	1.69E-05	0
NOTCH1	chr9	139396485	.	C	G	nonsynonymous_SNV	9q34.3	2/4	3008	0	0	0	0	0	0
NOTCH1	chr9	139399128	rs372830543	C	T	nonsynonymous_SNV	9q34.3	1/2	5013	2.00E-04	0	6.57E-04	0	0	0
NOTCH1	chr9	139399922	rs776133530	C	T	nonsynonymous_SNV	9q34.3	3/3	5098	0	0	0	0	3.40E-05	0
NOTCH1	chr9	139399975	rs374352922	G	A	nonsynonymous_SNV	9q34.3	1/2	4001	0	0	2.36E-04	0	2.57E-05	0
NOTCH1	chr9	139402753	.	G	T	nonsynonymous_SNV	9q34.3	1/1	1084	0	0	0	0	0	0
NOTCH1	chr9	139405670	rs369259434	C	T	nonsynonymous_SNV	9q34.3	2/2	2006	0	0	2.43E-04	0	4.26E-05	0
NOTCH1	chr9	139407931	rs587778559	CATT	C	nonframeshift_deletion	9q34.3	3/3	3076	0	0	0	0	2.00E-04	1.00E-04
NOTCH1	chr9	139409753	rs576030298	G	A	nonsynonymous_SNV	9q34.3	1/2	1070	0	0	0	0	3.37E-05	0
PRKDC	chr8	48690287	rs771401110	G	A	nonsynonymous_SNV	8q11.21	1/4; 1/2	3008; 4058	0	0	0	0	8.31E-06	1.00E-04
PRKDC	chr8	48691219	rs772389987	C	T	nonsynonymous_SNV	8q11.21	1/2	4047	0	0	0	0	0	0
PRKDC	chr8	48694958	rs547031184	C	T	nonsynonymous_SNV	8q11.21	1/2	3069	3.99E-04	0.002	0	0	3.00E-04	0.0037
PRKDC	chr8	48701739	rs772972344	C	G	nonsynonymous_SNV	8q11.21	1/1	1098	0	0	0	0	8.35E-06	0
PRKDC	chr8	48713488	rs752730508	T	C	nonsynonymous_SNV	8q11.21	2/3	1038	0	0	0	0	7.57E-05	0.001
PRKDC	chr8	48731966	rs373275113	G	C	nonsynonymous_SNV	8q11.21	1/2	4113	2.00E-04	0.001	0	0	3.32E-05	5.00E-04
PRKDC	chr8	48734290	rs544832472	G	A	nonsynonymous_SNV	8q11.21	2/2	3002	2.00E-04	0.001	0	0	2.00E-04	0.0031
PRKDC	chr8	48739338	rs185741285	C	T	nonsynonymous_SNV	8q11.21	2/2	4097	0	0	0	0	2.23E-05	0
PRKDC	chr8	48739421	.	T	A	nonsynonymous_SNV	8q11.21	1/2	1094	0	0	0	0	0	0
PRKDC	chr8	48749063	rs201726098	C	T	nonsynonymous_SNV	8q11.21	3/3	6014	2.00E-04	0	2.59E-04	0	2.00E-04	4.00E-04
PRKDC	chr8	48751805	rs763783256	T	A	nonsynonymous_SNV	8q11.21	1/1	4044	0	0	0	0	1.90E-05	3.00E-04
PRKDC	chr8	48761785	rs768108353	G	A	nonsynonymous_SNV	8q11.21	1/1	1057	0	0	0	0	4.97E-05	3.00E-04
PRKDC	chr8	48765247	rs753393942	A	G	nonsynonymous_SNV	8q11.21	1/3	4031	0	0	0	0	1.00E-04	0.0021
PRKDC	chr8	48792078	.	T	G	nonsynonymous_SNV	8q11.21	2/2	1003	0	0	0	0	0	0
PRKDC	chr8	48794579	rs56182356	G	C	nonsynonymous_SNV	8q11.21	1/3	3056	0	0	0	0	1.00E-04	3.00E-04

PRKDC	chr8	48809736	rs182548912	C	T	nonsynonymous_SNV	8q11.21	2/2; 1/3	5092; 6015	5.99E-04	0.003	0	0	1.00E-04	0.0019
PRKDC	chr8	48825111	.	A	C	nonsynonymous_SNV	8q11.21	2/2	2018	0	0	0	0	0	0
PRKDC	chr8	48830931	rs202216147	T	C	nonsynonymous_SNV	8q11.21	2/2	5012	0	0	0	0	0	0
PRKDC	chr8	48848381	rs760725135	A	G	nonsynonymous_SNV	8q11.21	1/3	5063	0	0	0	0	8.28E-06	1.00E-04
RAPGEF3	chr12	48131365	.	TCGGGA<br>GAGG	T	nonframeshift_deletion	12q13.11	3/3	5098	0	0	0	0	0	0
RAPGEF3	chr12	48131445	rs559675833	G	A	nonsynonymous_SNV	12q13.11	1/3	1038	2.00E-04	0.001	0	0	5.46E-05	5.00E-04
RAPGEF3	chr12	48133955	rs767312809	C	T	nonsynonymous_SNV	12q13.11	2/2	4113	0	0	0	0	2.74E-05	1.00E-04
RAPGEF3	chr12	48137823	rs767993156	T	C	nonsynonymous_SNV	12q13.11	1/2; 1/1	1085; 4033	0	0	0	0	2.65E-05	1.00E-04
RAPGEF3	chr12	48141583	rs749232919	C	T	nonsynonymous_SNV	12q13.11	2/3; 1/2	3049; 3050	0	0	0	0	4.15E-05	5.00E-04
RAPGEF3	chr12	48143184	rs767963161	G	A	nonsynonymous_SNV	12q13.11	3/3	1062	0	0	0	0	1.65E-05	0
RAPGEF3	chr12	48151717	.	G	A	nonsynonymous_SNV	12q13.11	2/2	1080	0	0	0	0	0	0
LFNG	chr7	2552292	rs768084728	G	A	nonsynonymous_SNV	7p22.3	1/1	6018	0	0	0	0	2.52E-05	3.00E-04
LFNG	chr7	2565127	rs377387325	C	T	nonsynonymous_SNV	7p22.3	3/3	6014	0	0	0	1.16E-04	0	0
MAML1	chr5	179192654	rs761942273	G	C	nonsynonymous_SNV	5q35.3	2/2	4084	0	0	0	0	5.77E-05	8.00E-04
MAML1	chr5	179193078	rs373506544	C	T	nonsynonymous_SNV	5q35.3	1/2; 1/2; 2/3	2018; 3083; 5040	2.00E-04	0.001	0	0	1.00E-04	0.0015
MAML1	chr5	179198178	rs368957465	C	T	nonsynonymous_SNV	5q35.3	2/2	3055	0	0	0	1.16E-04	5.77E-05	1.00E-04
MFNG	chr22	37870647	rs375750639	A	G	nonsynonymous_SNV	22q13.1	1/4	1026	0	0	0	1.16E-04	0	0
MFNG	chr22	37870660	rs574644668	T	C	nonsynonymous_SNV	22q13.1	1/1	4044	2.00E-04	0.001	0	0	6.64E-05	8.00E-04
MFNG	chr22	37875510	.	T	G	nonsynonymous_SNV	22q13.1	2/2	4113	0	0	0	0	0	0
MFNG	chr22	37875518	rs148692414	G	A	nonsynonymous_SNV	22q13.1	2/2	3063						
PSEN2	chr1	227071476	rs769031756	G	A	nonsynonymous_SNV	1q42.13	1/2	3097	0	0	0	0	8.25E-06	0
PSEN2	chr1	227075798	rs533813519	C	A	nonsynonymous_SNV	1q42.13	2/2	5092	2.00E-04	0.001	0	0	2.00E-04	0.0023
PSEN2	chr1	227077760	.	A	G	nonsynonymous_SNV	1q42.13	2/2	3002	0	0	0	0	0	0
PSEN2	chr1	227081825	.	T	C	nonsynonymous_SNV	1q42.13	2/3	3076	0	0	0	0	0	0

Ref, referent; Var, variant; KG, Thousand Genomes; AF, allele frequency; EAS, East Asians; ESP, Exome Sequencing Project; AA, African American; EA, European American; ExAC, Exome Aggregation Consortium

Supplemental Table 4: List of Variants Observed for Candidate Genes (KMT2C & MST1R) Identified in Two Published Whole Exome Sequencing Studies of NPC

Gene	Chrom.	Cytoband	Position	rsID	Ref	Var	Change observed	Protein Change	CADD Score	KG_AF	KG_EAS_AF	ESP_AA_AF	ESP_EA_AF	ExAC_ALL	ExAC_EAS	(No. NPC cases/obligate carriers (OC) with variant)/(total # NPC cases + OC sequenced)	ID of family(ies) in which variant observed
KMT2C	chr7	7q36.1	151845120	rs747747526	T	C	nonsynonymous_SNV	K4631R	24.9	0	0	0	0	8.91E-06	0.0001	1/1	4033
KMT2C	chr7	7q36.1	151853340	rs550819504	G	A	nonsynonymous_SNV	A3921V	32	0.0002	0.001	0	0	4.943E-05	0.0007	1/2; 1/2	1048; 3064
KMT2C	chr7	7q36.1	151855958	rs750387168	T	C	nonsynonymous_SNV	H3887R	18.21	0	0	0	0	2.471E-05	0.0001	2/2	4113
KMT2C	chr7	7q36.1	151859929	rs758091920	T	C	nonsynonymous_SNV	H3578R	5.661	0	0	0	0	2.471E-05	0.0003	1/2	5078
KMT2C	chr7	7q36.1	151878268	.	C	T	nonsynonymous_SNV	R2226K	26.4	0	0	0	0	0	0	2/2	4105
KMT2C	chr7	7q36.1	151884872	rs199796552	T	C	nonsynonymous_SNV	H1574R	23	0.0002	0.001	0	0	0.0002	0.0027	2/2; 1/2	1081; 3007
KMT2C	chr7	7q36.1	151927406	rs770115906	C	T	nonsynonymous_SNV	V924M	27.8	0	0	0	0	6.888E-05	0.0008	2/2	1076
KMT2C	chr7	7q36.1	151945043	rs763508651	C	A	nonsynonymous_SNV	G826C	28.6	0	0	0	0	1.648E-05	0.0002	1/1	1047
KMT2C	chr7	7q36.1	151945297	rs201735706	A	G	nonsynonymous_SNV	M741T	5.293	0.000998	0.004	0	0.000116	0.0002	0.0031	1/3; 1/2	1038; 5102
KMT2C	chr7	7q36.1	151970840	rs780548283	C	T	nonsynonymous_SNV	S321N	21.6	0	0	0	0	4.944E-05	0.0007	1/2; 2/3; 1/2	1070; 4013; 5004
KMT2C	chr7	7q36.1	152055717	rs530632312	T	A	nonsynonymous_SNV	M69L	23.5	0.0002	0.001	0	0	5.766E-05	0.0007	1/3	5041
KMT2C	chr7	7q36.1	152132801	rs768439100	G	A	nonsynonymous_SNV	A24V	21.6	0	0	0	0	0	0	1/3	5098
MST1R	chr3	3p21.31	49924740	rs746465044	T	A	stoploss	null	21.3	0	0	0	0	8.31E-06	0.0001	1/2	6040
MST1R	chr3	3p21.31	49936067	.	G	A	nonsynonymous_SNV	R535C	17.55	0	0	0	0	0	0	2/2	1081
MST1R	chr3	3p21.31	49939976	rs35924402	C	T	nonsynonymous_SNV	G356D	8.943	0	0	0	0	0.0001	0.0017	3/3	2007
MST1R	chr3	3p21.31	49940231	rs762985749	T	A	nonsynonymous_SNV	D271V	8.184	0	0	0	0	8.266E-06	0.0001	2/2	5096
